# A gene-based risk score model for predicting recurrence-free survival in patients with hepatocellular carcinoma

**DOI:** 10.1186/s12885-020-07692-6

**Published:** 2021-01-05

**Authors:** Wenhua Wang, Lingchen Wang, Xinsheng Xie, Yehong Yan, Yue Li, Quqin Lu

**Affiliations:** 1 Jiangxi Provincial Key Laboratory of Preventive Medicine, Nanchang University, Nanchang, 330006 Jiangxi China; 2 Department of Biostatistics and Epidemiology, School of Public Health, Nanchang University, Nanchang, 330006 Jiangxi China; 3 Center for Experimental Medicine, The First Affiliated Hospital of Nanchang University, Nanchang, 330006 Jiangxi China; 4 Department of General Surgery, The First Affiliated Hospital of Nanchang University, Nanchang, 330006 Jiangxi China

**Keywords:** TCGA, Hepatocellular carcinoma, Recurrence-free survival, Risk score, Prognostic model

## Abstract

**Background:**

Hepatocellular carcinoma (HCC) remains the most frequent liver cancer, accounting for approximately 90% of primary liver cancers worldwide. The recurrence-free survival (RFS) of HCC patients is a critical factor in devising a personal treatment plan. Thus, it is necessary to accurately forecast the prognosis of HCC patients in clinical practice.

**Methods:**

Using The Cancer Genome Atlas (TCGA) dataset, we identified genes associated with RFS. A robust likelihood-based survival modeling approach was used to select the best genes for the prognostic model. Then, the GSE76427 dataset was used to evaluate the prognostic model’s effectiveness.

**Results:**

We identified 1331 differentially expressed genes associated with RFS. Seven of these genes were selected to generate the prognostic model. The validation in both the TCGA cohort and GEO cohort demonstrated that the 7-gene prognostic model can predict the RFS of HCC patients. Meanwhile, the results of the multivariate Cox regression analysis showed that the 7-gene risk score model could function as an independent prognostic factor. In addition, according to the time-dependent ROC curve, the 7-gene risk score model performed better in predicting the RFS of the training set and the external validation dataset than the classical TNM staging and BCLC. Furthermore, these seven genes were found to be related to the occurrence and development of liver cancer by exploring three other databases.

**Conclusion:**

Our study identified a seven-gene signature for HCC RFS prediction that can be used as a novel and convenient prognostic tool. These seven genes might be potential target genes for metabolic therapy and the treatment of HCC.

## Background

In 2018, liver cancer remained among the top six prevalent carcinomas. There were 841,080 new patients, and 781,631 patients died of liver cancer according to the Global Cancer Statistics [1, 2]. Hepatocellular carcinoma (HCC) is the most frequent liver cancer, accounting for approximately 90% of primary liver cancers [3]. Currently, Hepatectomy and Radiofrequency ablation are the main two ways to treat HCC [4, 5]. Despite the continuous development of medical technology, the outcome of many patients who receive treatment and the prognosis of liver cancer remain poor with a 2-year recurrence rate of 76.9% [6–8]. And many studies have shown that HCC is the most difficult to cure cancer, and because of this, HCC has been described as a “chemoresistant” tumor [9]. Because of this, the prognosis of HCC is poor. The recurrence-free survival (RFS) of HCC patients is a critical factor in devising a personal treatment plan [10]. Thus, it is necessary to accurately forecast HCC patients’ prognosis to improve the prognosis of HCC. Most previous studies constructed prognostic models using the Tumor-Node-Metastasis (TNM) staging system to assess the prognosis of HCC patients [11]. However, the TNM staging system does not predict the prognosis of HCC. Therefore, it is important to develop a reliable tool for clinicians to predict the prognosis of patients with HCC.

Given the remarkable advances in high-throughput technologies, the development of The Cancer Genome Atlas (TCGA) (https://portal.gdc.cancer.gov/) and the intergovernmental Gene Expression Omnibus (GEO) (https://www.ncbi.nlm.nih.gov/gds) database provides an abundance of high-quality information regarding HCC [12]. Hence, it is urgent to develop methods to identify reliable therapeutic gene targets that could enable earlier prognostic evaluation and better therapeutic strategies [13]. Therefore, we considered whether we could build a gene-based risk score model [14]. Our goal was to generate simple and effective prognostic tools based on several genes and other factors that may affect RFS [13, 15]. Using the TCGA dataset, we selected 7 genes by robust likelihood-based survival modeling and built a risk score system [16, 17]. We used an independent dataset (GSE76427) to validate the effectiveness of the risk score system and demonstrate that its clinical value in predicting RFS in HCC patients is better than that of the TNM staging system.

## Methods

### Data collection and survival analyses

First, we downloaded gene expression profiles and clinical information from The Cancer Genome Atlas-liver hepatocellular carcinoma (TCGA-LIHC) dataset, which included 334 HCC samples [18]. We used GSE76427, which contained the gene expression and clinical information of 115 HCC samples, as the validation group. The samples in TCGA-LIHC and GSE76427 that met the following inclusion criteria were included in this study: all samples had mRNA sequencing data and clinical information related to RFS [19].

### Identification of genes associated with RFS

The raw count data were normalized with a log(a + 1) transformation. Then, using the “survfit” function in the “survival” package, we plotted Kaplan-Meier curves for the high and low expression groups of each gene. A log rank test with a *p*-value less than 0.05 was considered statistically significant [20].

### Enrichment analysis of GO functions and KEGG pathways

For the selected genes, we used WebGestalt (http://bioinfo.vanderbilt.edu/webgestalt) based on Gene Ontology (GO) functions and the Kyoto Encyclopedia of Genes and Genomes (KEGG) to understand the biological significance of the identified genes [21].

### Identification of the best genes for modeling

A robust likelihood-based survival approach was used to identify the best genes for modeling after determining the genes associated with RFS [22]. We used the “rbsurv” package in R to complete this modeling process.

### Construction and validation of the risk score system

A multivariate Cox regression analysis and “rbsurv” analysis were performed to identify the genes related to RFS and construct the prognostic gene signature. The “survivalROC” package in R was used to investigate the time-dependent prognostic value. The optimal cut-off values based on ROC curves were obtained to classify the patients into low-risk groups and high-risk groups. A calibration curve and the concordance index (C-index) were used to evaluate the risk score system.

### External validation of the risk score system

We calculated the risk score in the GSE76427 dataset. Then, the AUCs of the 12-month, 15-month, and 18-month RFS and Kaplan-Meier curves were used to verify the risk score system. A calibration curve was used to validate the risk score system. In addition, the prognosis-related genes included in the risk score system were verified at the protein level by using The Human Protein Atlas database. The CBioPortal for cancer genomics was used to study genetic alterations in the risk score system [23].

### Statistical analysis

The statistical tests were performed using R software and SPSS. Univariate and multivariate Cox regression analyses were performed using a forward stepwise procedure. A *p*-value less than 0.05 was considered statistically significant [23].

## Results

### Acquisition of the gene expression and clinical data

We downloaded the TCGA-LIHC dataset from The Cancer Genome Atlas (http://portal.gdc.cancer.gov/). The TCGA-LIHC dataset included 334 samples, 308 patients received hepatectomy, and the remaining 26 patients received radiofrequency ablation, and all samples included data regarding the RFS time and censoring status. The GSE76427 dataset was downloaded from the Gene Expression Omnibus database (http://www.ncbi.nlm.nih.gov/gov/). The GSE76427 dataset included 115 samples from HCC patients, but 7 patients had missing information regarding the RFS time and censoring status. Thus, 108 samples were included in this study, all 115 patients received hepatectomy. The median RFS times in the TCGA and GSE76427 series were 390 and 252 days, respectively, and the two datasets contained clinical information, such as gender, age, and the TNM stage.

### Genes associated with RFS

**Fig. 1:**
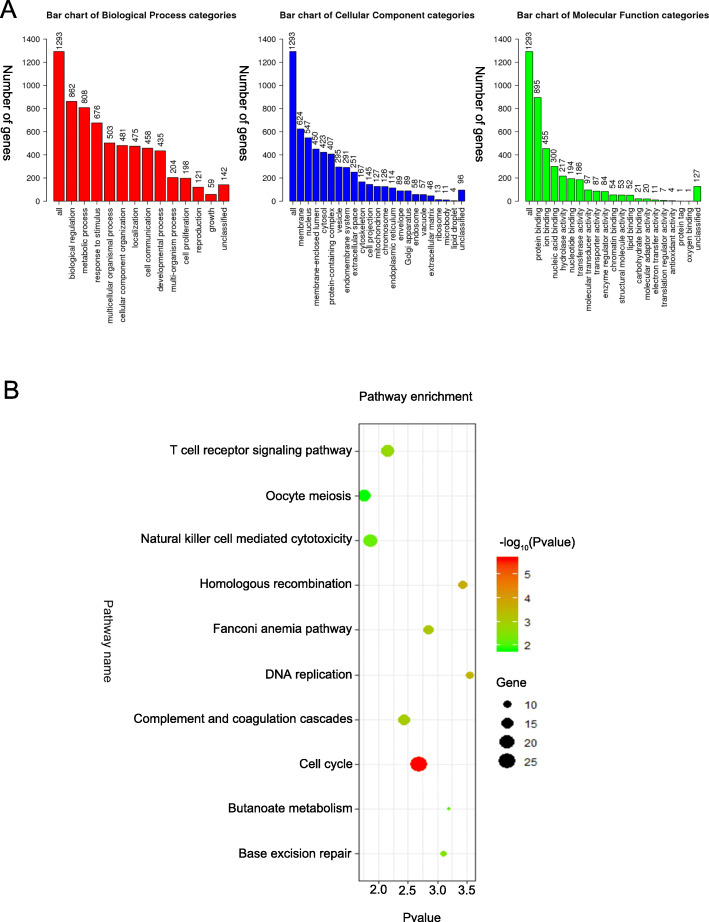
GO functional and KEGG pathway analyses. **a** Summary of the differentially expressed genes and GO pathway enrichment. Red, blue, and green bars represent the biological process, cellular component, and molecular function categories, respectively. The height of the bar represents the number of differentially expressed genes observed in each category. **b** The top 10 pathways of genes associated with RFS

We used the “survfit” function in the “survival” package and found 1331 genes associated with RFS. Then, to explore the genetic biological implications, we analyzed the 1331 genes through Gene Ontology (GO) functional and Kyoto Encyclopedia of Genes and Genomes (KEGG) pathway analyses. As shown in Fig. 1, in the KEGG analysis, we found that these genes are enriched in signaling pathways, such as the cell cycle, homologous recombination, DNA replication, the Fanconi anemia pathway, complement and coagulation cascades, and the T cell receptor signaling pathway.

### Construction of the prognostic model in TCGA-LIHC

Then, “rbsurv” was used to identify seven genes to construct the risk score system. The seven genes included in the system were TTK protein kinase (TTK), chromosome 16 open reading frame 54 (C16orf54), phosphoribosyl pyrophosphate amido transferase (PPAT), CD3e molecule associated protein (CD3EAP), solute carrier organic anion transporter family member 2A1 (SLCO2A1), acetyl-CoA acetyltransferase 1 (ACAT1), and growth-arrest specific 2 like 3 (GAS2L3) (Table 1).

**Table 1:** The best genes predicting recurrence-free survival of hepatocellular carcinoma patients

Gene symbol	nloglik	AIC	Select
TTK	808.79	1619.59	*
C16orf105	797.58	1599.16	*
PPAT	791.22	1588.43	*
CD3EAP	788.83	1585.66	*
SLCO2A1	787.91	1585.83	*
ACAT1	786.25	1584.50	*
GAS2L3	784.91	1583.83	*
SH2D5	784.84	1585.68	
ATP8A2	784.75	1587.50	
PABPC5	784.74	1589.49	

*Gene selected for the risk score

The risk score was calculated with the following formula: risk score = (− 0.038)*expression of TTK+(− 0.357)*expression of C16orf54 + 0.634*expression of PPAT+ 0.221*expression of CD3EAP+(− 0.076)*expression of SLCO2A1 + (− 0.184)*expression of ACAT1 + 0.277*expression of GAS2L3.

**Fig. 2:**
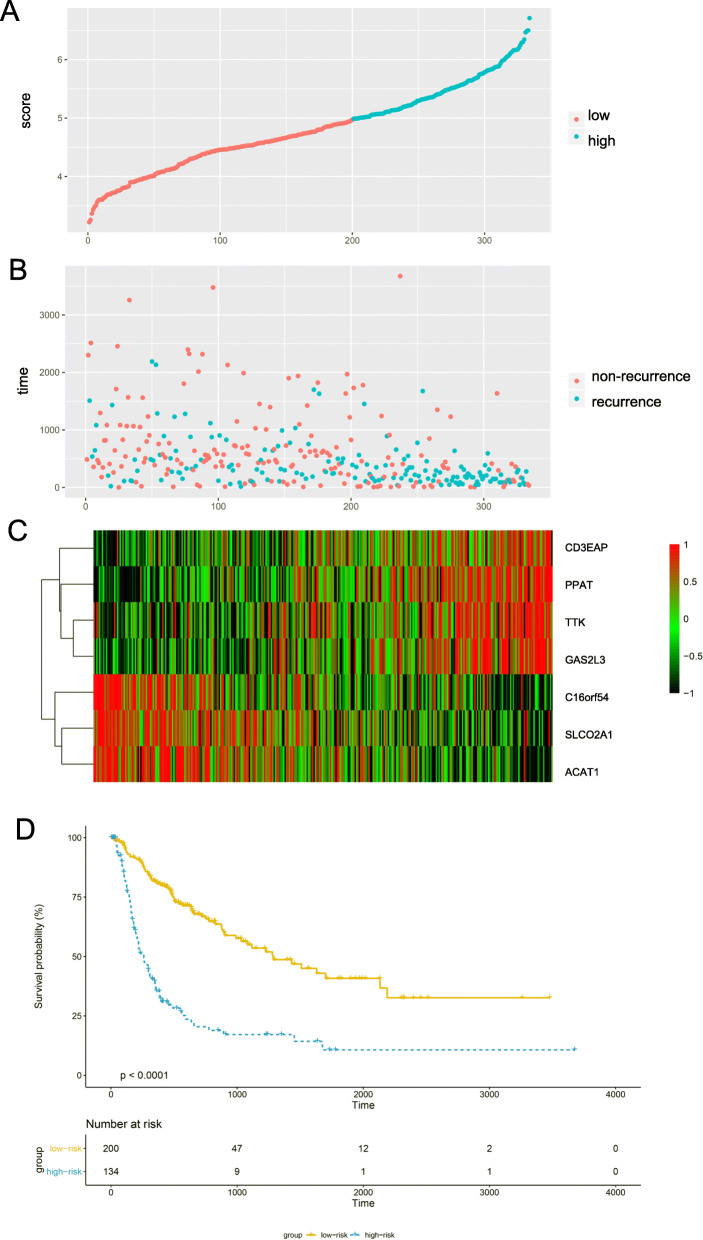
Analysis of the seven-gene signature of HCC in TCGA dataset. **a** Risk score of each patient; **b** The RFS time and RFS status of the HCC patients; **c** the expression levels of TTK, C16orf105, PPAT, CD3EAP, SLCO2A1, ACAT1 and GAS2L3 in the signature; Kaplan-Meier analysis of the TCGA dataset; **d** The Kaplan-Meier curve for the risk score model in TCGA dataset

In total, 334 patients were divided into two groups (134 high-risk patients and 200 low-risk patients) using a cut-off of 4.9798 for the risk score. Furthermore, the survival curve revealed that the RFS in the high-risk group was significantly poorer than that in the low-risk group (*p* < 0.0001; Fig. 2).

### Validation of the prognostic model in GSE76427

**Fig. 3:**
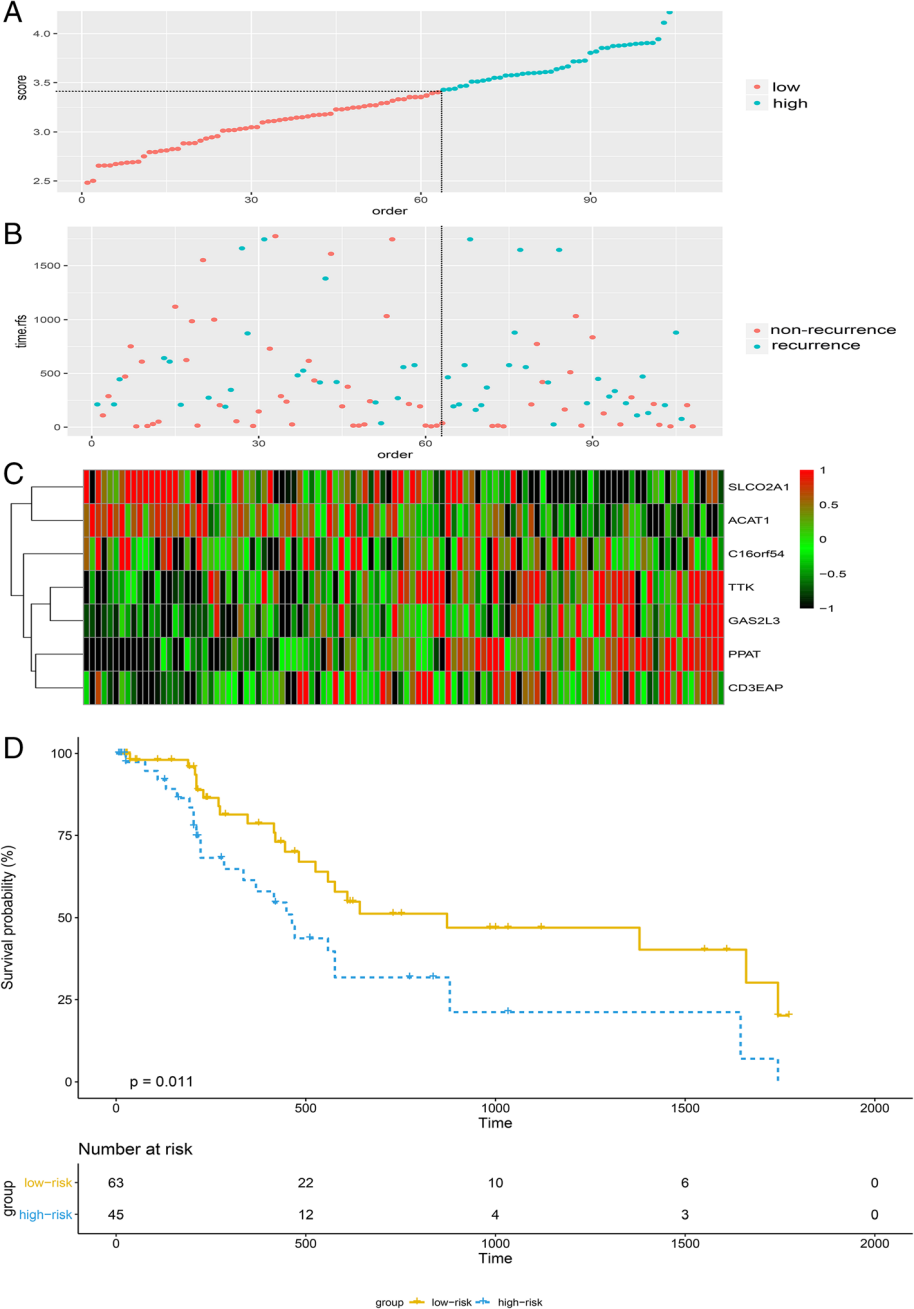
Analysis of the seven-gene signature of HCC in GEO dataset. **a** risk score of each patient; **b** The RFS time and RFS status of the HCC patients; **c** The expression levels of TTK, C16orf105, PPAT, CD3EAP, SLCO2A1, ACAT1 and GAS2L3 in the signature; Kaplan-Meier analysis of the GSE76427 dataset; **d** The Kaplan-Meier curve for the risk score model in GEO dataset

We validated the risk score system in the GSE76427 cohort. In total, 108 patients were divided into two groups (45 high-risk patients and 63 low-risk patients) using a cut-off of 3.4144 for the risk score. Furthermore, the survival curve revealed that the RFS in the high-risk group was significantly poorer than that in the low-risk group (*p* = 0.011; Fig. 3). In summary, these results indicate that the prognostic model has moderate sensitivity and specificity.

### Association between the prognostic model and the clinical characteristics of the patients

While assessing the correlation between the seven-gene signature and the clinical characteristics of the HCC patients, we found that a high risk score was significantly correlated with the TNM stage (*p* < 0.001), grade (*p* = 0.001), and AFP (*p* = 0.014), but was not significantly associated with the gender, age, BMI, or Child-Pugh score of the patients with HCC (Table 2). In GSE76427, the results showed that the 7-gene signature was not significantly associated with gender, age, BCLC (Barcelona Clinic Liver Cancer) or the TNM stage (Table 3).

**Table 2:** Characteristics of HCC patients in TCGA-LIHC dataset

	7-gene signature		The chi-square test	Univariate cox regression	
Variables Score	Low-risk (200)	High-risk (134)	*p* value	HR 3.607	*p* value < 0.001
**Gender**			0.330	0.975	0.879
Male	140	87			
female	60	47			
**Age (years)**			0.785	1.048	0.769
< 60	91	63			
≥ 60	109	71			
**BMI (kg/m**^**2**^**)**			0.061	0.900	0.509
< 25	91	75			
≥ 25	109	59			
**TNM**			< 0.001	1.680	< 0.001
I	123	44			
II	44	39			
III	31	50			
IV	2	1			
**Grade**			0.001	1.112	0.515
1 + 2	139	68			
3 + 4	61	64			
NA	0	2			
**AFP (ng/ml)**			0.014	0.976	0.913
< 300	134	63			
≥ 300	31	30			
NA	35	41			
**Child-Pugh score**			0.082	1.202	0.581
A	136	68			
B-C	10	11			
NA	56	55

**Table 3:** Characteristics of HCC patients in GSE 76427 dataset

	7-gene signature		The chi-square test	Univariate cox regression	
Variables Score	Low-risk (63)	High-risk (45)	*p* value	HR 2.047	*p* value 0.014
**gender**			0.374	0.609	0.208
Male	11	11			
female	52	34			
**Age (years)**			0.161	1.048	0.769
< 60	21	21			
≥ 60	42	24			
**TNM**			0.877	1.267	0.191
I	36	16			
II	15	19			
III	10	9			
IV	2	1			
**BCLC**			0.877	1.112	0.515
0	2	2			
A	41	30			
B	16	9			
C	4	4			

### Independent prognostic role of the prognostic gene signature

**Fig. 4:**
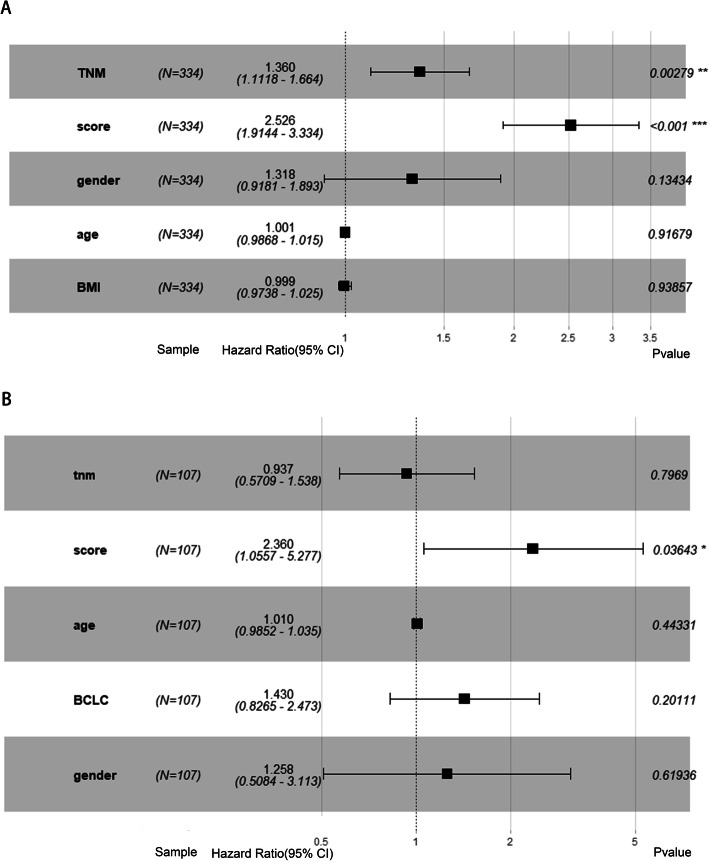
Multivariate Cox regression analysis. **a** Multivariate Cox regression analysis of the TCGA dataset. **b** Multivariate Cox regression analysis of the GSE76427 dataset

Moreover, the results of the multivariate Cox regression analysis showed that the TNM stage (HR = 1.680, *p* < 0.001) and our prognostic model (HR = 3.607, *p* < 0.001) were both independent factors of RFS among the 334 TCGA-LIHC patients. However, among the 108 patients in the GSE76427 cohort, the TNM stage was not an independent prognostic factor for RFS [24]. The prognostic model (HR = 2.407, *p* = 0.014) was also an independent factor for RFS (Fig. 4). In addition, we performed univariate and multivariate Cox regression with other well-known pathological factors such as vascular invasion and hepatic virus infection status in TCGA-LIHC hepatectomized patients. The results prove that our prognostic model is an independent prognostic factor as well (Table 4).

**Table 4:** Univariate and multivariate Cox regression in TCGA-LIHC hepatectomized patients

Variables	Univariate Cox regression			Multivariate Cox regression		
	HR	95% CI	***p*** value	HR	95% CI	***p*** value
risk score	2.788	2.174–3.574	< 0.001	2.501	1.660–3.376	< 0.001
vascular invasion	1.509	1.139–2.000	0.004	1.439	0.949–2.183	0.087
hepatic virus infection status	1.170	0.760–1.800	0.476	1.050	0.625–1.765	0.854

### Comparison of the TNM stage model and BCLC model

**Fig. 5:**
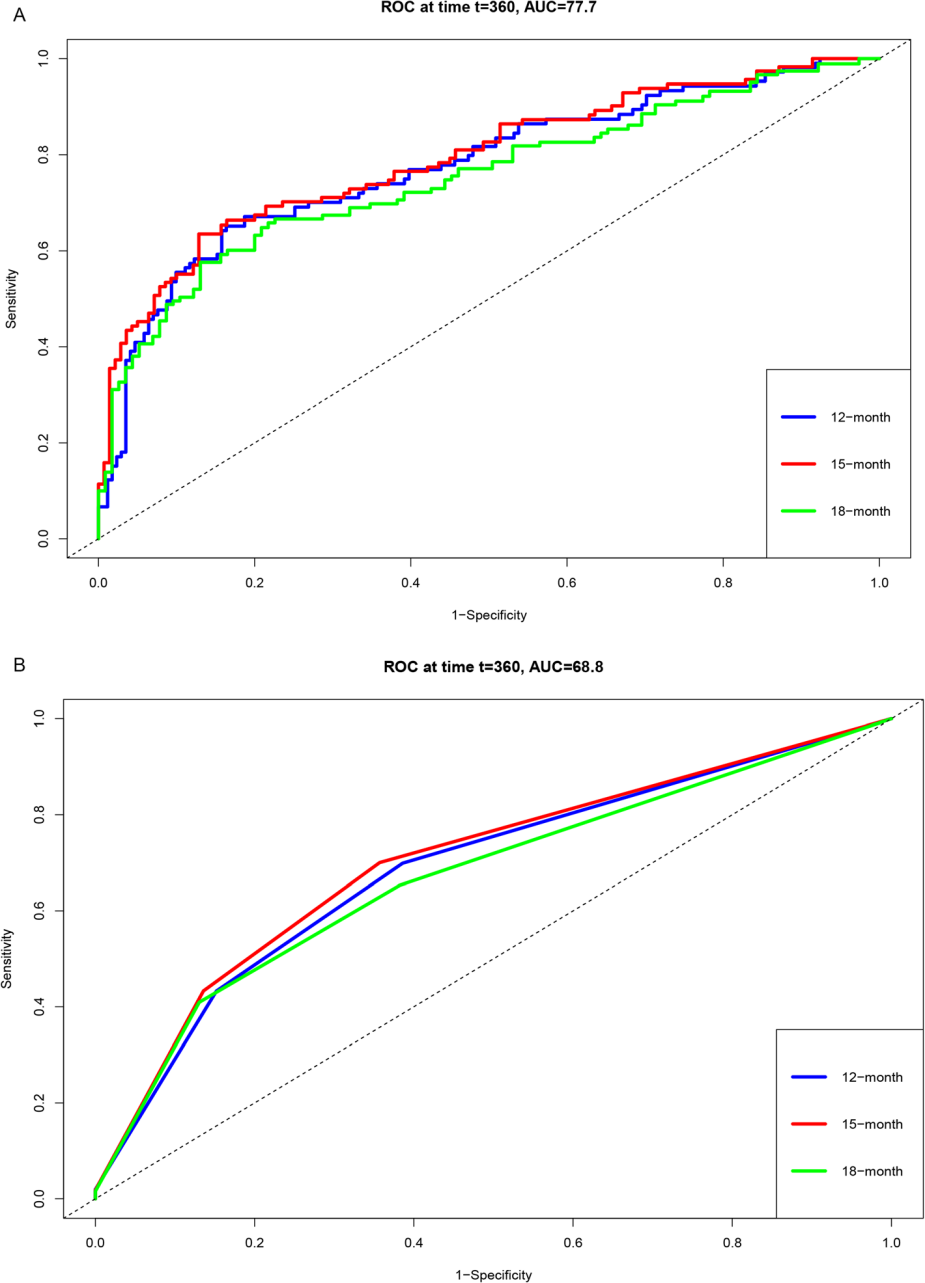
Validation of the risk score predicting RFS for HCC patients in TCGA-LIHC dataset. **a** The prognostic model’s AUCs of the 12-, 15-, and 18-month RFS in the TCGA-LIHC dataset. **b** The TNM stage model’s AUCs of the 12-, 15-, and 18-month RFS in the TCGA-LIHC dataset

**Fig. 6:**
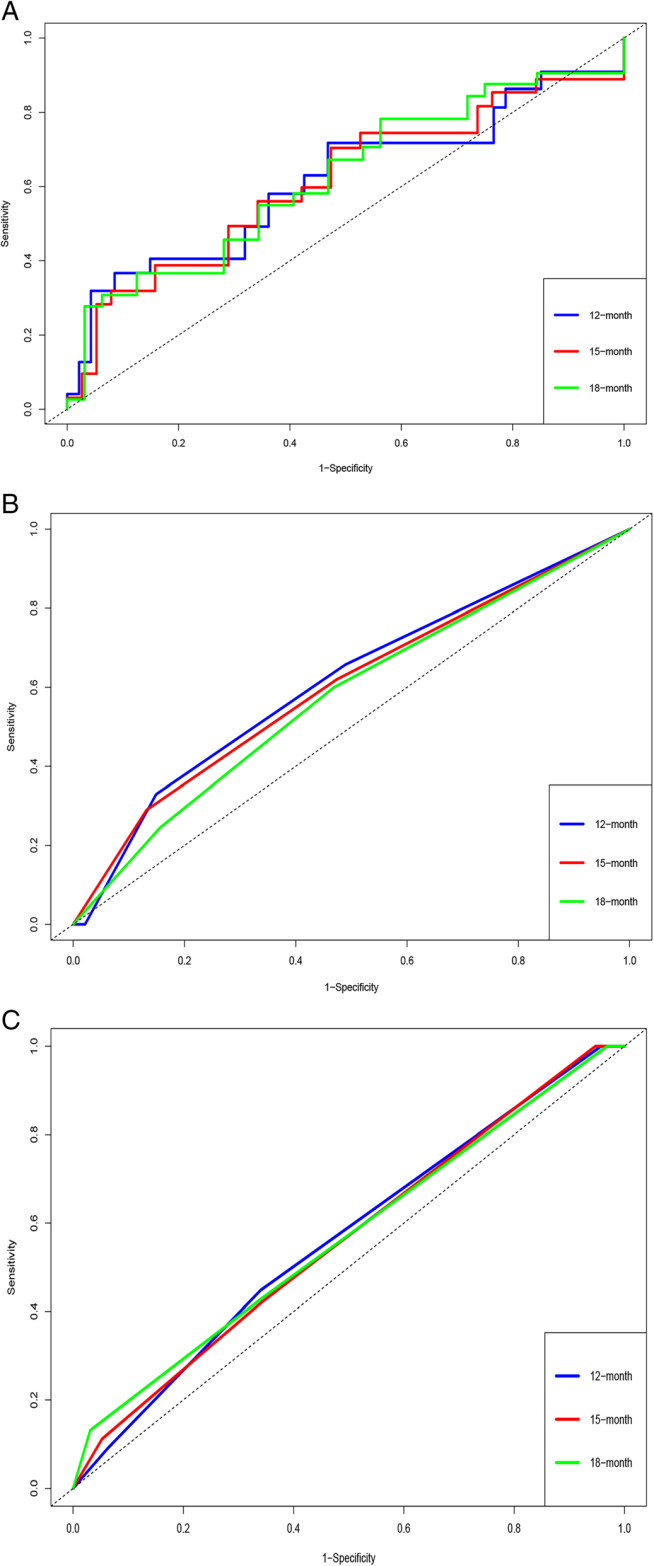
Validation of the risk score predicting RFS for HCC patients in GSE76427 dataset. **a** The prognostic model’s AUCs of the 12-, 15-, and 18- month RFS in the GSE76427 dataset. **b** The TNM stage model’s AUCs of the 12-, 15-, and 18-month RFS in the GSE76427 dataset. **c** The BCLC model’s AUCs of the 12-, 15-, and 18-month RFS in the GSE76427 dataset

To compare the accuracy of the prognostic model and the TNM model, we calculated the AUCs of the 12-month, 15-month, and 18-month RFS. In the TCGA-LIHC dataset, the prognostic model’s AUCs of the 12-month, 15-month, and 18-month RFS were 0.7768, 0.7934, and 0.7529, and the TNM model’s AUCs of the 12-month, 15-month, and 18-month RFS were 0.6884, 0.7026, and 0.6721, respectively (Fig. 5). In the GSE76427 dataset, the prognostic model’s AUCs of the 12-month, 15-month, and 18-month RFS were 0.6159, 0.6118, and 0.6217, and the TNM model’s AUCs of the 12-month, 15-month, and 18-month RFS were 0.6122, 0.6009, and 0.5762, respectively. In addition, the BCLC model’s AUCs of the 12-month, 15-month, and 18-month RFS were 0.5669, 0.5627, and 0.5684, respectively (Table 5). Overall, our prognostic model showed a benefit in predicting the RFS, which might help doctors with targeted treatment (Fig. 6).

**Table 5:** Comparison of the prognostic model with the TNM and BCLC model

Model	TNM model	BCLC model	Prognostic model
TCGA-LIHC			
12-month AUC	0.6884 (0.6272–0.7496)		0.7768 (0.7180–0.8356)
15-month AUC	0.7026 (0.6416–0.7636)		0.7934 (0.7367–0.8501)
18-mouth AUC	0.6721 (0.6086–07356)		0.7529 (0.6905–0.8153)
GSE76427			
12-month AUC	0.6122 (0.4733–0.7511)	0.5669 (0.4408–0.6931)	0.6159 (0.4596–0.7722)
15-month AUC	0.6009 (0.4692–0.7326)	0.5627 (0.4400–0.6853)	0.6118 (0.4679–0.7575)
18-mouth AUC	0.5762 (0.4453–0.7072)	0.5684 (0.4458–0.6910)	0.6217 (0.4828–0.7605)

### Development of the calibration curve

**Fig. 7:**
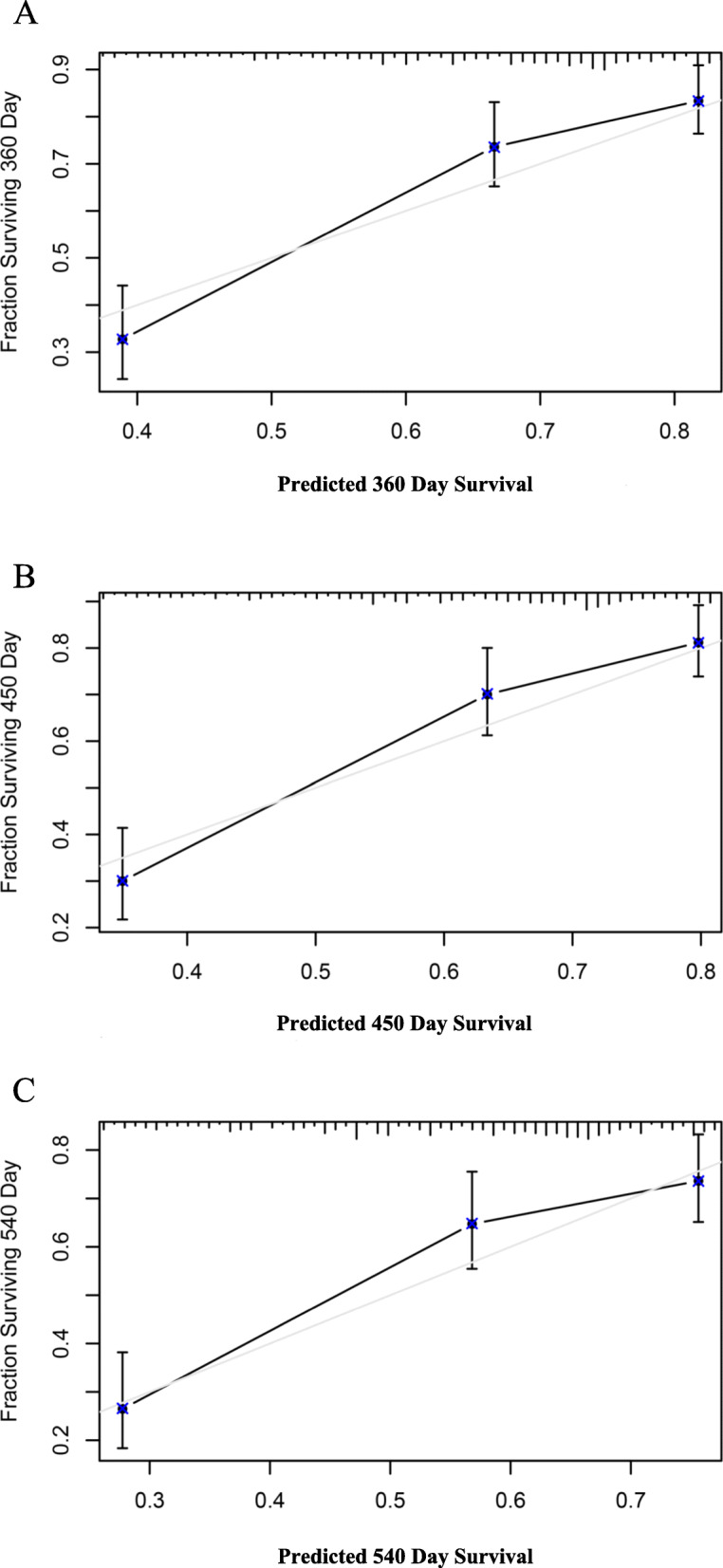
Calibration curve for the 12-month, 15-month, and 18-month periods in the TCGA-LIHC dataset. **a** The prognostic model was used to generate a calibration curve for the 12-month RFS prediction. **b** The prognostic model was used to generate a calibration curve for the 15-month RFS prediction. **c** The prognostic model was used to generate a calibration curve for the 18-month RFS prediction

**Fig. 8:**
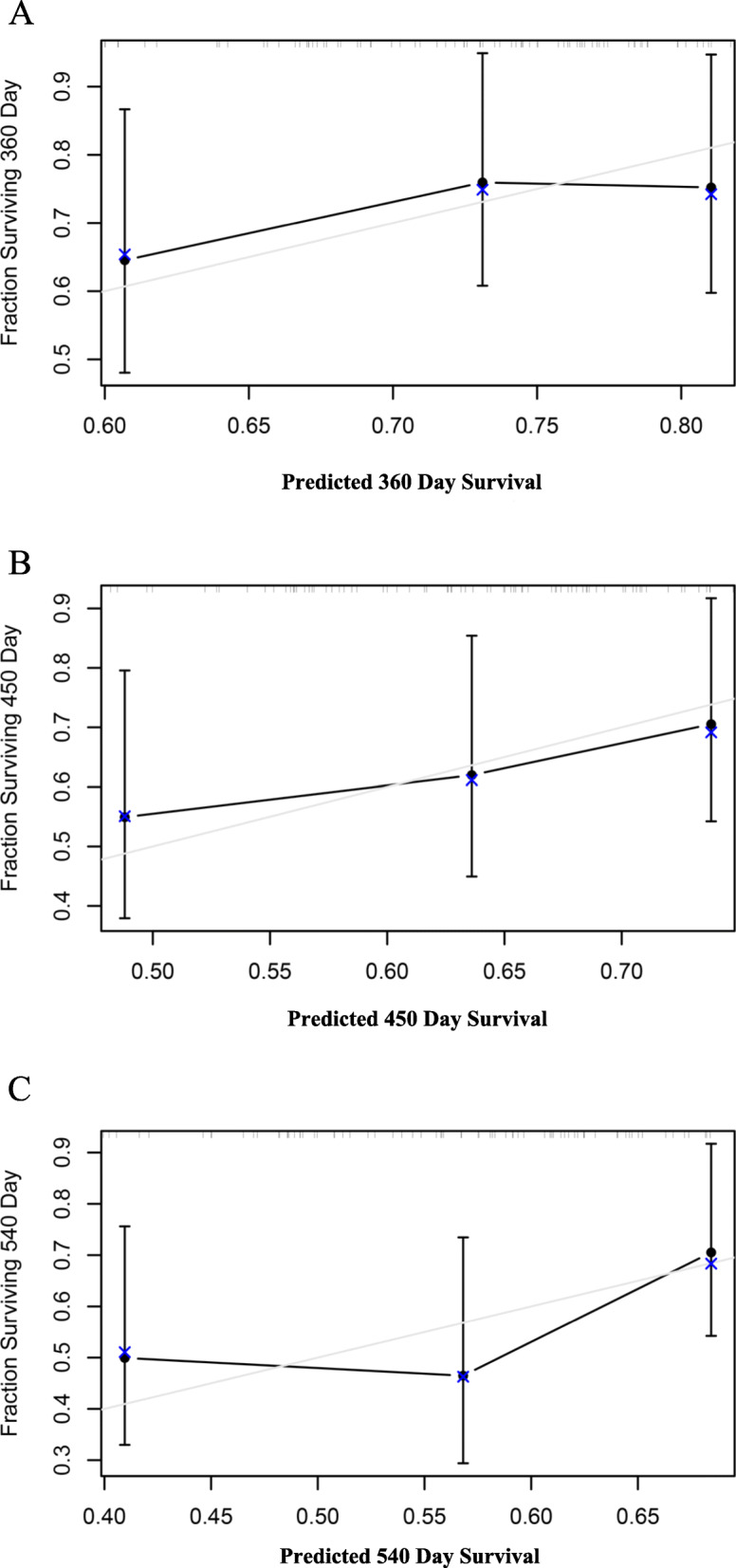
Calibration curve for the 12-month, 15-month, and 18-month periods in the GSE76427 dataset. **a** The prognostic model was used to generate a calibration curve for the 12-month RFS prediction. **b** The prognostic model was used to generate a calibration curve for the 15-month RFS prediction. **c** The prognostic model was used to generate a calibration curve for the 18-month RFS prediction

We calculated the C-index and drew calibration curves for the 12-, 15- and 18-month survival predictions to evaluate the calibration in the TCGA-LIHC dataset and the GSE76427 dataset. The C-index of the TCGA-LIHC dataset and GSE76427 dataset was 0.717 and 0.647, respectively, as shown in Figs. 7 and 8.

### External validation in an online database

**Fig. 9:**
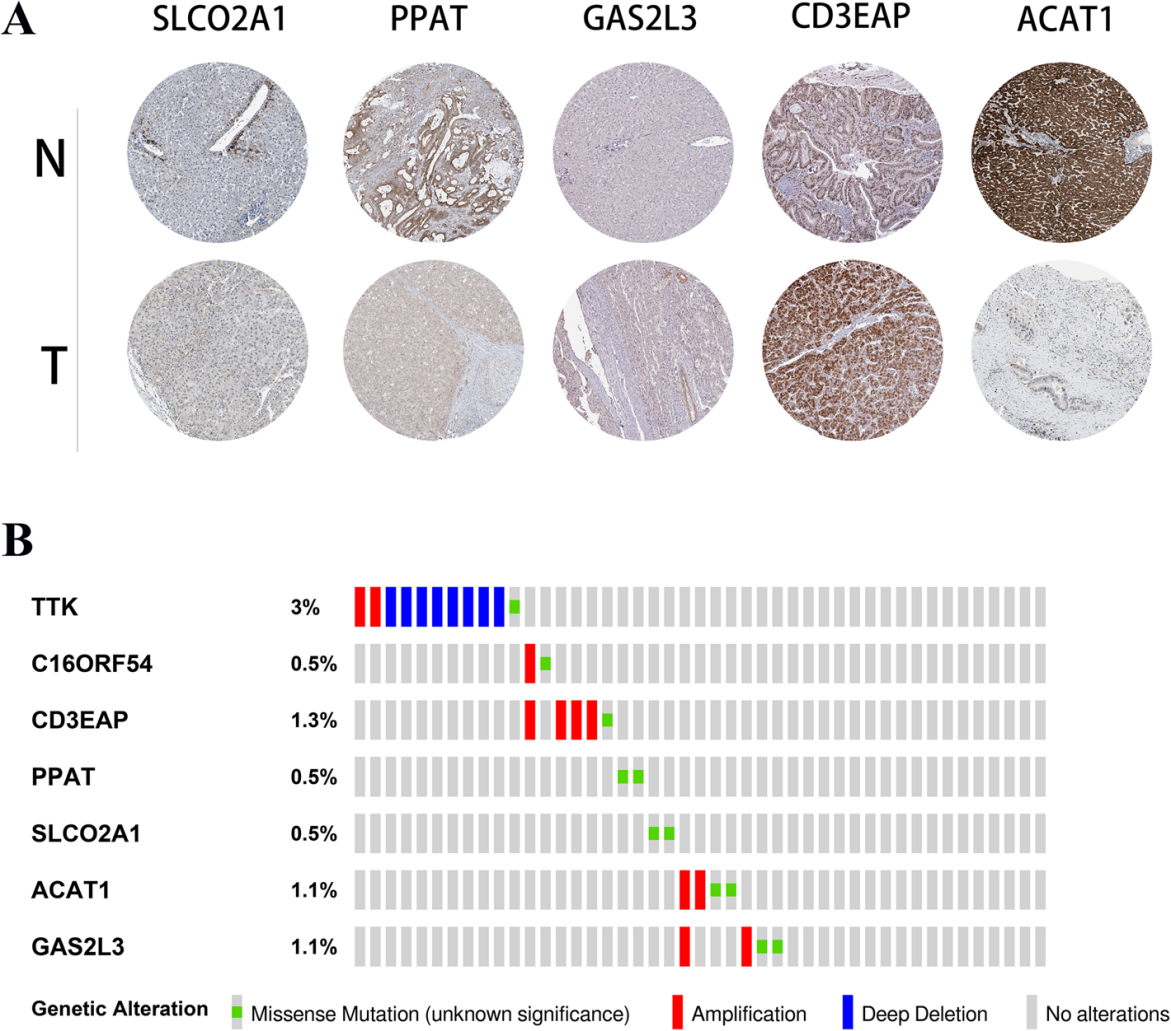
External validation in online databases. **a** Representative protein expression levels of the seven genes in HCC and normal liver tissue. **b** Genetic alterations of the seven genes

**Fig. 10:**
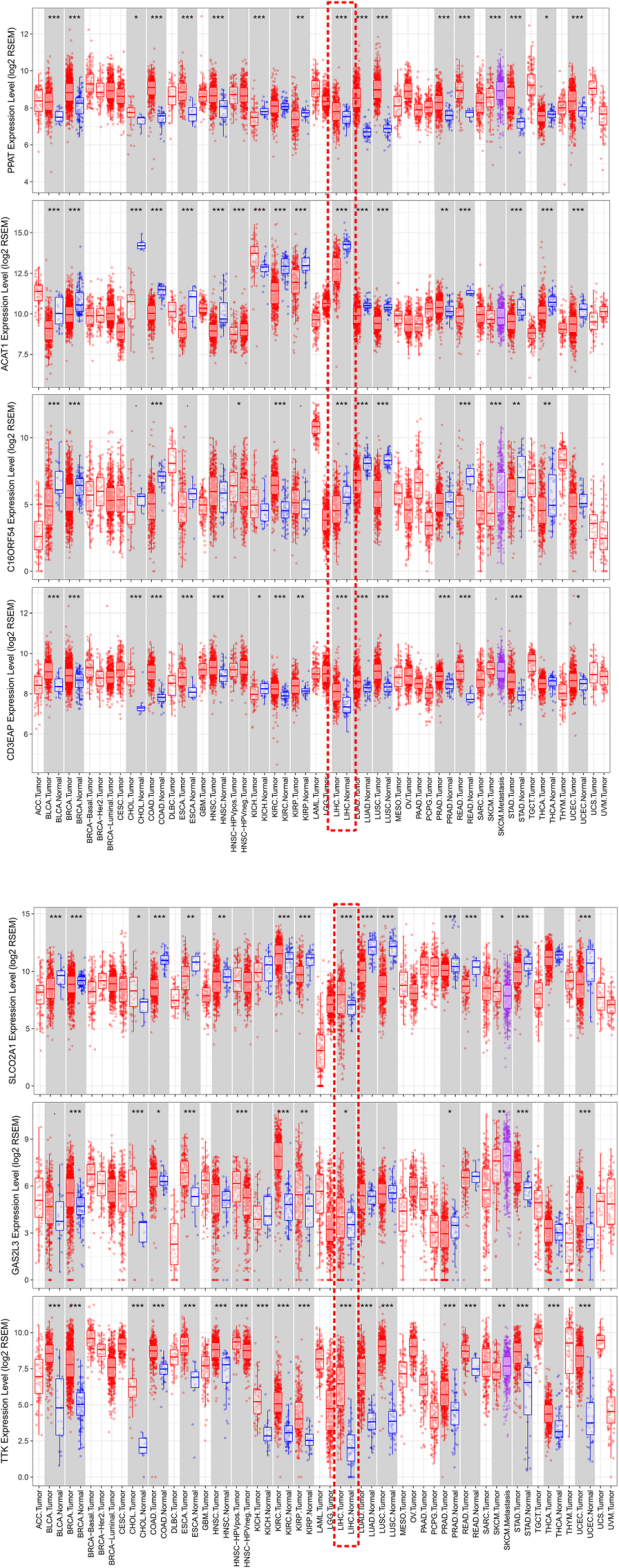
Expression levels of the seven genes in different cancers

The representative protein expression levels of SLCO2A1, PPAT, GAS2L3, CD3EAP, and ACAT1 were explored in the Human Protein Profiles. Then, we explored the TTK, C16orf54, PPAT, CD3EAP, SLCO2A1, ACAT1, and GAS2L3 genes in the CBioPortal for cancer genomics. TTK exhibited the most frequent genetic alterations (3%), and deep deletion was the most frequent alteration. The second most altered gene was CD3EAP (1.3%), and the most frequent alterations were amplification mutations (Fig. 9). The expression levels of the seven genes in different cancers are shown in Fig. 10. In summary, the aberrant expression of these seven genes may explain some of the abnormal expression of these genes.

## Discussion

In this study, we developed a risk score based on seven genes that has the ability to predict the probability of RFS in HCC patients and is more accurate than clinical indicators. Using this model, we can identify patients with HCC who have a higher risk of recurrence, indicating that these patients need more attention. In the TCGA-LIHC dataset, in total, 1331 genes were found to be associated with RFS in HCC patients. In the KEGG analysis, we found that the 1331 genes were enriched in signaling pathways, such as the cell cycle, homologous recombination, DNA replication, the Fanconi anemia pathway, complement and coagulation cascades, and the T cell receptor signaling pathway. This finding suggests that the 7-gene signature might affect the RFS of HCC patients through these pathways. Then, we selected the best 7 genes to develop the risk score model as follows: TTK, C16orf105, PPAT, CD3EAP, SLCO2A1, ACAT1, and GAS2L3. Additionally, our study showed that the TNM staging system is not an accurate indicator for the prediction of RFS in HCC patients, which is consistent with the results of other studies. According to the prognostic model, we divided the patients into low- and high-risk groups, which exhibited significant differences in RFS. This result indicated that the prognostic model could be used as a conventional tool for the prediction of the RFS of HCC patients.

The prognostic model was validated using another independent dataset, i.e., GSE76427. The area under the curve revealed the ability of the prognostic model to differentiate the patients’ prognoses; the survival curve represents the survival of the high-risk group, which had a worse prognosis compared with that of the low-risk group. These findings demonstrate that the prognostic model has the ability to forecast RFS in HCC patients.

Most of the seven genes in our prognostic model have been reported to be involved in cancer. The TTK protein levels differ in human liver cancer between liver cancer cells and adjacent noncancerous liver cells [25]. This study also tested the utility of TTK-targeted inhibition and demonstrated its therapeutic potential in an experimental model of liver cancer in vivo. Furthermore, our study demonstrated its effectiveness and incorporated it into the prognostic model. PPAT, which a member of the purine/pyrimidine phosphoribosyl transferase family, regulates pyruvate kinase activity and cell proliferation and invasion and is a biomarker of lung adenocarcinoma. Acetyl-CoA acetyltransferase (ACAT) was recently reported to be elevated in human cancer cell lines [16]. ACAT1 exhibits acetyltransferase activity and can acetylate pyruvate dehydrogenase (PDH), which affects tumor growth [26].

In other scholars’ prognostic analysis of HCC, CD3EAP is also a predictor, suggesting that CD3EAP is an important predictor of HCC prognosis, but the function of CD3EAP is not completely clear [27]. The function of GAS2L3 is still unknown, and GAS2L3 may be involved in mediating the absorption and clearance of prostaglandins, but its function in liver cancer has not been reported [19]. Moreover, SLCO2A1 and C16orf105 have not been reported in previous HCC studies, indicating that these genes may be potential factors in the treatment of HCC. Understanding the function of these genes may promote the development of HCC treatment.

However, despite the potential substantial clinical significance of our results, this study still has some limitations. One limitation is that although the calibration curve performance and AUC value were excellent in the validation group, multicenter clinical application is needed to further evaluate the external utility of the prognostic model [28]. Second, only 1331 genes were defined as genes associated with RFS and evaluated for the prognostic model construction. Some important genes could have been excluded before building the prognostic model [29]. In addition, knowledge regarding signaling pathways is urgently needed to reveal the functions of these genes in HCC. Finally, other well-known pathological factors, such as vascular invasion and hepatic virus infection status, should be key topics of our further studies. After collecting clinical tumor tissues with pathological information, we will find a way to combine our risk score with these clinical characteristics. Meanwhile, we have realized that many studies showed that different surgical methods had an impact on the prognosis of HCC patients. We will pay attention to distinguishing surgical methods when collecting clinical cases and compare the difference in the predictive effect of risk score on RFS in patients receiving different surgical methods in our future study.

## Conclusions

In conclusion, we developed and validated a prognostic model for the prediction of the RFS probability of HCC patients. The simple prognostic model has the ability to predict RFS and could be a useful tool for doctors conducting an evaluation of HCC and selecting treatment plans for HCC patients.

## Data Availability

The gene expression profiles and clinical information datasets downloaded from The Cancer Genome Atlas (TCGA-LIHC)(https://portal.gdc.cancer.gov) and the Gene Expression Omnibus (GEO)(https://www.ncbi.nlm.nih.gov), accession numbers: GSE76427. Genetic alterations was retrieved from the cBioPortal website (http://www.cbioportal.org/).

## Abbreviations

HCC: Hepatocellular carcinoma
RFS: Recurrence-free survival
TCGA: The Cancer Genome Atlas
GEO: The intergovernmental Gene Expression Omnibus
ROC: Receiver Operating Characteristic curve
TNM: Tumor Node Metastasis
BCLC: Barcelona Clinic Liver Cancer
TCGA-LIHC: The Cancer Genome Atlas-liver hepatocellular carcinoma
GO: Gene Ontology
KEGG: Kyoto Encyclopedia of Genes and Genomes
C-index: Concordance index
AUC: Area Under Curve
BMI: Body mass index
AFP: alpha fetoprotein
HR: Hazard Ratio
NA: Not available

## References

[1] Siegel RL, Miller KD, Jemal A (2019). Cancer statistics, 2019. CA Cancer J Clin.

[2] Torre LA, Bray F, Siegel RL, Ferlay J, Lortet-Tieulent J, Jemal A (2015). Global cancer statistics, 2012. CA Cancer J Clin.

[3] Li G, Xu W, Zhang L, Liu T, Jin G, Song J (2019). Development and validation of a CIMP-associated prognostic model for hepatocellular carcinoma. EBioMedicine.

[4] Facciorusso A, Serviddio G, Muscatiello N (2016). Transarterial radioembolization vs chemoembolization for hepatocarcinoma patients: a systematic review and meta-analysis. World J Hepatol.

[5] Rognoni C, Ciani O, Sommariva S, Facciorusso A, Tarricone R, Bhoori S (2016). Trans-arterial radioembolization in intermediate-advanced hepatocellular carcinoma: systematic review and meta-analyses. Oncotarget.

[6] Chun YH, Kim SU, Park JY, Kim DY, Han KH, Chon CY (2011). Prognostic value of the 7th edition of the AJCC staging system as a clinical staging system in patients with hepatocellular carcinoma. Eur J Cancer.

[7] Facciorusso A (2013). The influence of diabetes in the pathogenesis and the clinical course of hepatocellular carcinoma: recent findings and new perspectives. Curr Diabetes Rev.

[8] Facciorusso A (2018). Drug-eluting beads transarterial chemoembolization for hepatocellular carcinoma: current state of the art. World J Gastroenterol.

[9] Cabral LKD, Tiribelli C, Sukowati CHC. Sorafenib resistance in hepatocellular carcinoma: the relevance of genetic heterogeneity. Cancers. 2020;12(6):1576. 10.3390/cancers12061576 PMC7352671 32549224

[10] Gu JX, Zhang X, Miao RC, Xiang XH, Fu YN, Zhang JY (2019). Six-long non-coding RNA signature predicts recurrence-free survival in hepatocellular carcinoma. World J Gastroenterol.

[11] Amin MB, Greene FL, Edge SB, Compton CC, Gershenwald JE, Brookland RK (2017). The eighth edition AJCC cancer staging manual: continuing to build a bridge from a population-based to a more "personalized" approach to cancer staging. CA Cancer J Clin.

[12] Liao X, Yang C, Huang R, Han C, Yu T, Huang K (2018). Identification of potential prognostic long non-coding RNA biomarkers for predicting survival in patients with hepatocellular carcinoma. Cell Physiol Biochem.

[13] Gao Z, Zhang D, Duan Y, Yan L, Fan Y, Fang Z (2019). A five-gene signature predicts overall survival of patients with papillary renal cell carcinoma. PLoS One.

[14] Chen SH, Wan QS, Zhou D, Wang T, Hu J, He YT (2019). A simple-to-use Nomogram for predicting the survival of early hepatocellular carcinoma patients. Front Oncol.

[15] Yuan SX, Yang F, Yang Y, Tao QF, Zhang J, Huang G (2012). Long noncoding RNA associated with microvascular invasion in hepatocellular carcinoma promotes angiogenesis and serves as a predictor for hepatocellular carcinoma patients' poor recurrence-free survival after hepatectomy. Hepatology.

[16] Goudarzi A (2019). The recent insights into the function of ACAT1: a possible anti-cancer therapeutic target. Life Sci.

[17] Lee JH, Jung S, Park WS, Choe EK, Kim E, Shin R (2019). Prognostic nomogram of hypoxia-related genes predicting overall survival of colorectal cancer-analysis of TCGA database. Sci Rep.

[18] Joyce S, Nour AM (2019). Blocking transmembrane219 protein signaling inhibits autophagy and restores normal cell death. PLoS One.

[19] Wang Y, Sun L, Li Z, Gao J, Ge S, Zhang C (2019). Hepatoid adenocarcinoma of the stomach: a unique subgroup with distinct clinicopathological and molecular features. Gastric Cancer.

[20] Liu GM, Zeng HD, Zhang CY, Xu JW (2019). Identification of a six-gene signature predicting overall survival for hepatocellular carcinoma. Cancer Cell Int.

[21] Wang L, Yan Z, He X, Zhang C, Yu H, Lu Q (2019). A 5-gene prognostic nomogram predicting survival probability of glioblastoma patients. Brain Behav.

[22] Luo D, Deng B, Weng M, Luo Z, Nie X (2018). A prognostic 4-lncRNA expression signature for lung squamous cell carcinoma. Artif Cells Nanomed Biotechnol.

[23] Liu GM, Xie WX, Zhang CY. Identification of a four-gene metabolic signature predicting overall survival for hepatocellular carcinoma. J Cell Physiology. 2019;235(2):1624-1636. 10.1002/jcp.29081 31309563

[24] Buti S, Karakiewicz PI, Bersanelli M, Capitanio U, Tian Z, Cortellini A (2019). Validation of the GRade, age, nodes and tumor (GRANT) score within the surveillance epidemiology and end results (SEER) database: a new tool to predict survival in surgically treated renal cell carcinoma patients. Sci Rep.

[25] Miao R, Wu Y, Zhang H, Zhou H, Sun X, Csizmadia E (2016). Utility of the dual-specificity protein kinase TTK as a therapeutic target for intrahepatic spread of liver cancer. Sci Rep.

[26] Chen L, Peng T, Luo Y, Zhou F, Wang G, Qian K (2019). ACAT1 and metabolism-related pathways are essential for the progression of clear cell renal cell carcinoma (ccRCC), as determined by co-expression network analysis. Front Oncol.

[27] Zhang G, Xue P, Cui S, Yu T, Xiao M, Zhang Q (2018). Different splicing isoforms of ERCC1 affect the expression of its overlapping genes CD3EAP and PPP1R13L, and indicate a potential application in non-small cell lung cancer treatment. Int J Oncol.

[28] Abdelnabi M, Almaghraby A, Saleh Y, Abd Elsamad S (2019). Hepatocellular carcinoma with a direct right atrial extension in an HCV patient previously treated with direct-acting antiviral therapy: a case report. Egypt Heart J.

[29] Abou-Alfa GK, Shi Q, Knox JJ, Kaubisch A, Niedzwiecki D, Posey J, et al. Assessment of treatment with Sorafenib plus doxorubicin vs Sorafenib alone in patients with advanced hepatocellular carcinoma: phase 3 CALGB 80802 randomized clinical trial. JAMA Oncology. 2019;5(11):1582-1588. 10.1001/jamaoncol.2019.2792 PMC6735405 31486832

